# Dilated common bile duct is commonly associated with main duct Intraductal Papillary Mucinous Neoplasm of the pancreas

**DOI:** 10.1186/s12876-024-03291-y

**Published:** 2024-06-17

**Authors:** Wisam Sbeit, Amir Shahin, Tawfik Khoury

**Affiliations:** 1 Department of Gastroenterology, Galilee Medical Center, Nahariya, Israel; 2 Faculty of Medicine in the Galilee, Bar-Ilan University, Safed, Israel

**Keywords:** Pancreas, Cysts, IPMN, Common bile duct, Dilatation

## Abstract

**Background:**

Dilatation of common bile duct (CBD) is mostly pathological and mainly occurs secondary to mechanical causes. We aimed to explore the prevalence of CBD dilatation in Intraductal Papillary Mucinous Neoplasms of the pancreas (IPMN) among patients referred to EUS.

**Methods:**

A retrospective study of all patients who had an EUS diagnosis of IPMN from 2011 to 2019 at Galilee Medical Center were extracted. Control group including patients with other types of pancreatic cysts.

**Results:**

Overall, 2400 patients were included in the study, of them 158 patients were diagnosed with pancreatic cysts, 117 patients (74%) diagnosed with IPMN (group A), and 41 patients (26%) diagnosed with other pancreatic cysts (group B). Univariate analysis showed significant association of IPMN (OR 3.8, 95% CI 1.3–11.5), resected gallbladder (GB) (OR 7.75, 95% CI 3.19–18.84), and age (OR 1, 95% CI 1.01–1.08) with CBD dilatation. Classifying IPMN to sub-groups using adjusted multivariate logistic regression analysis, only main duct-IPMN (MD-IPMN) significantly correlated with CBD dilatation compared to branch duct (BD)-IPMN and mixed type-IPMN (OR 19.6, 95% CI 4.57–83.33, OR 16.3, 95% CI 3.02–88.08).

**Conclusion:**

MD-IPMN was significantly correlated with dilated CBD. Assessment of the pancreas is warranted in encountered cases of dilated CBD without obvious mechanical cause.

## Introduction

With the increased availability and use of cross-sectional imaging, diverse incidental findings are now diagnosed frequently including pancreatic cysts which are encountered in over 2% of computed tomography and magnetic resonance imaging examinations; a frequency that rise with age [1, 2]. About 38% of them are Intraductal Papillary Mucinous Neoplasms (IPMN) [3]. Typically, they are formed by intraductal neoplastic proliferations of columnar, mucin-containing cells with a variable degree of papillary formation and cyst formation [4, 5]. IPMN grows from cells of the main duct or the branch ducts of the pancreas, and accordingly are classified into three types based on involvement of pancreatic ducts: main duct IPMN (MD-IPMN), branch duct IPMN (BD-IPMN) and mixed type IPMN (M-IPMN) [6, 7]. MD-IPMN affects any segment or diffusely the main pancreatic duct and is more aggressive with higher malignant potential than BD-IPMN [8]. On the other hand, BD-IPMN usually arises in the uncinate process, but may involve the tail of the pancreas, but it has a lower malignant potential than MD-IPMN [9, 10]. Mixed duct type behaves like MD-IPMN [7, 9]. The adult common bile duct (CBD) accepted upper normal diameter measurement is up to 6 mm. Although its width is supposed to be stable in the absence of resistance to bile flow, however it increases with age but remains within normal limits and increases post cholecystectomy [10]. A large study by Perret RS et al. reached the conclusion that in the overwhelming majority of patients 60 years of age or older the mean diameter of the CBD remained ≤ 6 mm [11]. A systematic review evaluating incidental CBD dilatation identified a cause in about 33% where the most common causes were CBD stone, chronic pancreatitis, and periampullary diverticulum. Coexisting dilatation of both bile and pancreatic ducts usually designated as double duct sign, suggests obstructing pancreatic disease, especially malignancy which may also be accompanied by obstructive jaundice [12]. Notably, CBD dilatation was reported in recent years to be an independent predictor of malignancy in IPMN [13]. Also a study seeking computed tomography features suggestive of malignant or invasive IPMN identified dilatation of CBD to be more common in malignant IPMN [14]. However, the prevalence of CBD dilatation in the different types of IPMN was not reported yet. A finding that may guide further investigation for CBD dilatation without evident mechanical cause. The aim of our study was to assess the prevalence of CBD dilatation with IPMN compared to other pancreatic cystic lesions, and to compare its association with IPMN sub-types (Branch duct, main duct, and mixed duct IPMN).

## Materials and methods

### Study design

We performed a case control retrospective study single center study, using the databases of Galilee Medical Center.

### Study population

All patients above 18 years old who were referred to endoscopic ultrasound (EUS) for further investigation of an episode of idiopathic pancreatitis and/or had cystic lesion in the pancreas according to abdominal cross-sectional imaging and who had EUS morphological diagnosis of pancreatic cysts from 1.1.2010 till 1.10.2019 were included in the study. Patients were excluded if they had hepato-pancreato-biliary malignancies, choledocholithiasis and obstructive jaundice. Extracted data included demographic variables (age, gender), smoking, personal or family history of malignancy, EUS findings, presence of gallbladder, CBD stones, CBD width by EUS and the morphological diagnosis.

### Intervention

All procedures were carried out via linear echoendoscope (Pentax-Japan), model 3870 and performed by endoscopist (WS) with more than 10 years’ experience in the field of Endoscopic Ultrasound. Patients were placed in the left lateral decubitus position and were sedated with intravenous midazolam and propofol according to the decision of the endoscopist.

### Outcome measures and definitions

#### 1. Diagnosis of pancreatic cystic lesions

The diagnosis of pancreatic cystic lesions in our study was based on two criteria, the morphological EUS criteria and the biochemical cyst analysis (amylase and Carcinoembryonic antigen (CEA)), as follows:

#### a. Morphologic sonographic characterization of pancreatic cyst

Four types of pancreatic cysts were reported in our study. The morphologic sonographic diagnosis of the pancreatic cysts in our study was made according to the following characteristics: (1) IPMNs: MD-IPMNs are characterized by pancreatic duct dilatation in EUS without obstructing lesion or stone, while branch duct type is characterized by cystic dilatation of branch ducts connected to the main pancreatic duct, whereas, mixed type represents a combination of both types [15]. (2) Mucinous cystic neoplasm (MCN): female dominancy, typically consists of multiple (less than 6 cm) macrocystic (bigger than 1–2 cm in diameter) locules, but may be unilocular, most commonly located in the body or tail of pancreas and characterized by cystic lesion not connected to the pancreatic ducts and in about 15% of cases contains pathognomonic peripheral wall calcification [15, 16]. (3) Serous cyst adenoma (SCA): focal lesions that may be located anywhere in the pancreas, usually consisting of multiple small cysts separated by septa, resembling a honeycomb with pathognomonic central calcification in up to 20% of cases [17], and (4) Pseudocyst (PC) – mostly extra pancreatic cyst with thin muddy-brown debris occurring in patient with history of moderate to severe pancreatitis or abdominal trauma [18].

#### b. Biochemical cyst fluid analysis

The cyst fluid was analyzed for amylase and CEA level. Unfortunately, reported sensitivities and specificities of chemical analyses have broad ranges, making interpretation difficult [19, 20]. The American society of gastrointestinal endoscopy (ASGE) guidelines recommend a cut-off level of CEA of > 192 ng/mL for pancreatic mucinous cysts (MCN and IPMN) and CEA < 5 ng/mL for serous cysts [18]. However, it doesn’t recommend a cut-off level for cyst amylase. Similarly, the World Gastroenterology Guidelines, report a CEA level > 192 ng/mL for mucinous cysts: IPMN and MCN (sensitivity 73%, specificity 84%) and < 5 ng/mL for serous cysts (sensitivity 100% and specificity 86%) (WGO Global Guideline Pancreatic Cystic Lesions 2019). For PC, The European Study Group on Cystic Tumors of the Pancreas guidelines reported that low level amylase may exclude pancreatic pseudocysts (amylase < 250 U/L; sensitivity 44%, specificity 98%) [21]. Therefore, in our study, the agreement between the morphologic sonographic diagnosis and the FNA cyst fluid analysis results were set as CEA > 192 ng/mL for mucinous cysts while CEA < 5 ng/mL for diagnosis of SCA and amylase > 250 unit/lit for PC.

#### 2. Diagnosis of CBD dilatation

CBD dilatation was defined as > 6 mm width by EUS examination.

### Statistical analysis

Descriptive statistic was used to compare patients with IPMN diagnosis and other pancreatic cystic lesions. Data was reported as mean ± standard deviation for quantitative continuous variables, and frequencies (percentages) for categorial variables. Univariate logistic regression was used to estimate odds ratio (OR) of groups (IPMN, other pancreatic cysts) and baseline factors on CBD dilatation, and multiple logistic regression analysis was used to adjust the OR of IPMN sub-groups vs. CBD dilatation by age and resected GB. Between group comparisons for groups were done using post-hoc testing in the logistic regression, with the Tukey-Kramer correction for multiple testing. The decision to include gallbladder and age in the model was based on clinical and statistical considerations. A threshold for statistical significance was set at a P value < 0.05. All analyses were performed by an experienced statistician using the statistical analysis software (SAS Vs 9.4 Copyright (c) 2016 by SAS Institute Inc., Cary, NC, USA).

## Results

### Demographics, baseline characteristics and endoscopic findings

**Fig. 1:**
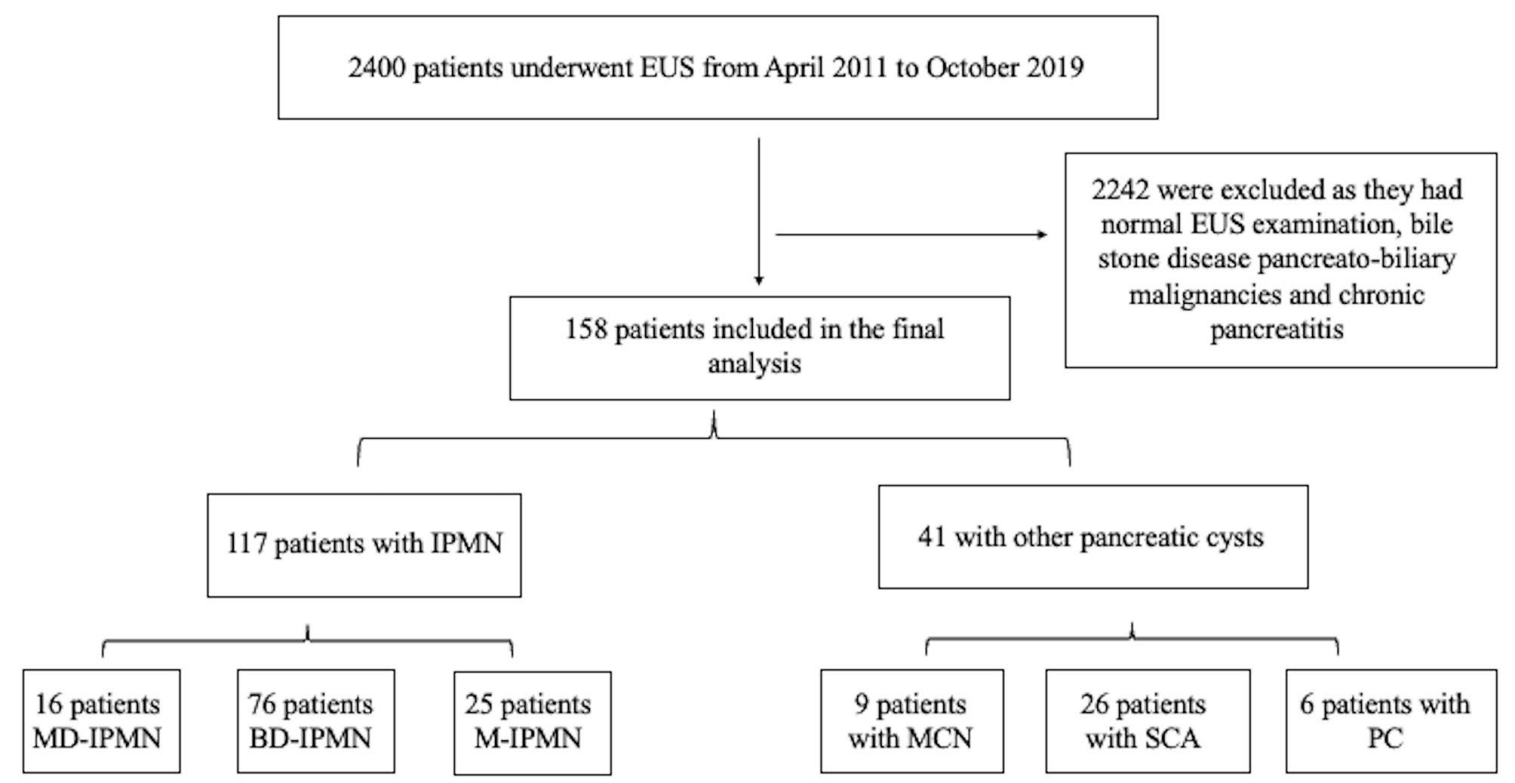
Demonstrating the flow chart of our study

Overall, 2400 patients were included in the study, of them, 2242 were excluded as: 1675 patients had gallstones disease, 343 patients had pancreato-biliary malignancies, and 224 patients had normal EUS examination, remaining with 158 patients who were diagnosed with pancreatic cystic lesions (16 patients had MD-IPMN, 76 patients had BD-IPMN, 25 patients had M-IPMN, 9 patients had MCN, 26 patients had SCA, and 6 patients had PC) according to the EUS diagnostic criteria mentioned above. Regarding the number of successful fluid collections, overall, 98 patients had FNA, and cyst amylase and CEA successfully obtained. Among the remaining patients who had not have FNA were 28 patients with BD-IPMN (21 patients had cyst size < 10 mm, and in 7 patients FNA aspirated very few and viscous fluid insufficient for biochemical analysis), 13 patients with MD-IPMN and 9 patients with M-IPMN (FNA was not attempted due to typical morphological appearance of IPMN, and maximal main pancreatic duct width up to 7 mm), 1 patient with MCN (EUS-FNA was not attempted due to intervening vessel), 9 patients with SCA (classic morphological appearance of microcystic adenoma). We identified 117 patients (74%) diagnosed with IPMN (group A) and 41 patients (26%) diagnosed with other pancreatic cysts (group B). Among group B, 9 patients were diagnosed with MCN (none of them progressed to adenocarcinoma at the time of diagnosis), 26 patients with SCA and 6 patients with PC (Fig. 1). The mean cyst levels of amylase and CEA were as follows: IPMN (amylase of 72,204 U/L, CEA of 1325 ng/mL), for MCN (amylase of 116 U/L, CEA of 1369 ng/mL), for SCA (amylase of 495 U/L, CEA of 5.4 ng/mL) and for PC (amylase of 110,045 U/L, CEA of 137 ng/mL). The most common cyst location in group A was in the head and in all pancreatic parts (head, body, and tail) in 26.5% each, while the most common location in group B was in the pancreatic head (39%). Thirty-four patients (29.1%) in group A had dilated CBD as compared to only 4 patients (9.7%) in group B. The mean CBD diameter in group A (IPMN group) was 7 ± 2.3 mm (range: 3–17 mm), as compared to 6.3 ± 1.1 mm (range: 6–10.7 mm) in group B (non-IPMN group, *P* = 0.01). Moreover, the CBD diameter in BD-IPMN, MD-IPMN, and M-IPMN was 6.3 ± 1.5 mm (range 3–7 mm), 9.3 ± 2.9 mm (range 7–16 mm), and 7.1 ± 2.5 mm (range 5.2–17 mm), respectively. Table 1 demonstrated the baseline characteristics, and the endoscopic findings, as well Table 2 demonstrates the baseline characteristics among the sub-types of the pancreatic cysts.

**Table 1:** Demonstrates the baseline characteristics and the morphologic endoscopic findings

	IPMN patients	Other pancreatic cysts	*P* value
Number of patients	117	41	-
Age (years), mean ± SD	74.6 ± 10.4	61.1 ± 14	***< 0.001***
Gender, N (%)			0.8
• Male • Female	49 (41.9) 68 (58.1)	18 (43.9) 23 (56.1)	
Family history of pancreatic cancer, N (%)	3 (2.6)	1 (2.4)	0.1
Smoking, N (%)	6 (5.1)	2 (4.9)	0.1
**Background liver diseases, N (%)**			
Cholestatic liver disease Other liver disease (HCV)	0 6 (5.1)	0 0	0.3
Personal history of cancer, N (%)	22 (18.8)	5 (12.2)	0.4
Alcohol consumption, N (%)	1 (0.9)	2 (4.9)	0.1
Gallbladder stone by EUS, N (%)	23 (19.7)	3 (7.3)	0.08
Signs of chronic pancreatitis, N (%)	2 (1.7)	2 (4.9)	0.2
Gallbladder in situ, N (%)	93 (80)	37 (90.2)	0.1
Cyst size by EUS (mm), mean ± SD	15.7 ± 10.4	26.6 ± 20.7	***< 0.001***
CBD dilation by EUS, N (%)	34 (29.1)	4 (9.7)	***0.01***
CBD diameter by EUS (mm), mean ± SD	7 ± 2.3 (3–17)	6.3 ± 1.1 (6-10.7)	***0.06***
Amylase level (U/L), mean	72,204	23,584	***0.03***
CEA level (ng/mL), mean	1325	441	***0.05***

**Table 2:** Demonstrates the baseline characteristics and the morphologic endoscopic findings among the cysts sub-types

	IPMN			Other cyst types		
	BD-IPMN	MD-IPMN	M-IPMN	MCN	SCA	PC
Number of patients	76	16	25	9	26	6
Age (years), mean ± SD	73.1 ± 10.6	73.9 ± 10.6	79.8 ± 8	65.4 ± 10.2	60.5 ± 15	57.2 ± 14.9
Gender, N (%)						
Male Female	26 (34.2) 50 (65.8)	7 (43.7) 9 (56.3)	16 (64) 9 (36)	4 5	9 17	5 1
Family history of pancreatic cancer, N (%)	2 (2.6)	0	0	0	0	1 (16.7)
Smoking, N (%)	0	1 (6.2)	5 (20)	0	0	2 (33.3)
**Background liver diseases, N (%)**						
Cholestatic liver disease Other liver disease (HCV)	0 5 (6.6)	0 0	0 1 (4)	0 0	0 0	0 0
Personal history of cancer, N (%)	16 (21)	2 (12.5)	4 (16)	2 (22.2)	2 (7.7)	1 (16.7)
Alcohol consumption, N (%)	1 (1.3)	0	0	0	0	2 (33.3)
Gallbladder stone by EUS, N (%)	15 (19.7)	4 (25)	4 (16)	2 (22.2)	1 (3.8)	0
Signs of chronic pancreatitis, N (%)	0	1 (6.2)	1 (4)	0	0	2 (33.3)
Gallbladder in situ, N (%)	64 (84.2)	10 (62.5)	19 (75)	6 (66.7)	26 (100)	5 (83.3)
CBD dilation by EUS, N (%)	14 (18.4)	13 (81.2)	7 (28)	3 (33.3)	1 (3.8)	0
CBD diameter by EUS (mm), mean ± SD	6.3 ± 1.5 (3–7)	9.3 ± 2.9 (7–16)	7.1 ± 2.5 (5.2–17)	7.1 ± 1.9 (6–10)	6.1 ± 0.7 (6–9)	6 (6)
Main pancreatic duct diameter by EUS (mm), mean ± SD	2 ± 0.4	5.5 ± 1.9	4.8 ± 1.5	2.1 ± 0.5	1.9 ± 0.3	3.5 ± 1.2
Amylase level (U/L), mean ± SD	38,808	230,839	149,952	116	495	110,045
CEA level (ng/mL), mean ± SD	1764	39.2	252	1369	5.4	137

### Univariate analysis of parameters associated with CBD dilatation

On univariate logistic regression analysis of baseline parameters associated with CBD dilatation, three parameters showed significant correlation with CBD dilatation including IPMN diagnosis (OR 3.8, 95% CI 1.3–11.5, *P* = 0.01), resected gallbladder (OR 7.75, 95% CI 3.19–18.84, *P* < 0.001) and age (OR 1, 95% CI 1.01–1.08, *P* = 0.006) ***(***Table 3***).*** Moreover, univariate logistic regression analysis of CBD dilatation among group B and IPMN sub-groups comparing the odds of each sub-type of IPMN to each other, and to the other pancreatic cyst types (group B), CBD dilatation was significantly higher in the MD-IPMN patients compared to the BD-IPMN and M-IPMN (OR 19.23, 95% CI 4.81–76.92, and OR 11.14, 95% CI 2.41–51.41), respectively. While there was no difference in CBD dilatation between BD-IPMN and M-IPMN (OR 0.58, 95% CI 0.2–1.66) ***(***Table 4***).***

**Table 3:** Univariate logistic regression of baseline parameters associated with CBD dilatation

Parameter	CBD dilatation (%)	Odds Ratio	95% Confidence Limit	*P* value
Age	-	1	1.01–1.08	***0.006***
IPMN diagnosis	29.1	3.8	1.3–11.5	***0.01***
Gender Male vs. Female	14.9/30.8	0.4	0.18–0.9	0.02
Family history of pancreatic cancer	50	1.34	0.15–11.76	0.79
Background liver disease	33.3	1.77	0.32–9.81	0.51
Personal history of cancer	37	2.18	0.9–5.27	0.08
Smoking	12.5	0.6	0.09–4.03	0.6
Alcohol	0	0.44	0.01–13.61	0.64
Gallbladder stone by EUS	30.8	1.54	0.61–3.88	0.36
Resected gallbladder	60.7	7.75	3.19–18.84	***< 0.001***

**Table 4:** Univariate logistic regression analysis of CBD dilatation among group B and IPMN sub-groups

Parameter	CBD dilatation (%)	Odds Ratio	95% Confidence Limit	Adjusted *P* value
Group A vs. group B	29.1 vs. 9.7	3.79	1.25–11.45	**< 0.0001**
MD-IPMN vs. group B	81.2 vs. 9.7	40	7.87–200	***< 0.0001***
BD-IPMN vs. group B	18.4 vs. 9.7	2.1	0.64–6.8	0.61
M-IPMN vs. group B	28 vs. 9.7	3.6	0.93–13.89	0.25
MD-IPMN vs. BD-IPMN	81.2 vs. 18.4	19.23	4.81–76.92	***0.0002***
MD-IPMN vs. M-IPMN	81.2 vs. 28	11.14	2.41–51.41	***0.01***
BD-IPMN vs. M-IPMN	18.4 vs. 28	0.58	0.2–1.66	0.74

*Post-Hoc testing in the univariate logistic regression, with the Tukey-Kramer correction for multiple testing*

### Multivariate logistic regression analysis of CBD dilatation among group B, and IPMN sub-groups

On multivariate logistic analysis, MD-IPMN remained significantly associated with CBD dilatation compared to group B, BD-IPMN and M-IPMN (OR 25.64, 95% CI 4.59-142.86, OR 19.6, 95% CI 4.57–83.33, and OR 16.3, 95% CI 3.02–88.08), respectively. While there was no difference between BD-IPMN compared to M-IPMN (OR 0.82, 95% CI 0.25–2.7), and between either BD-IPMN or M-IPMN compared to group B (Table 5).

**Table 5:** Multivariate logistic regression analysis of CBD dilation among group B and IPMN sub-groups

Parameter	CBD dilation (%)	Odds Ratio	95% Confidence Limit	Adjusted *P* value
MD-IPMN vs. group B	81.2 vs. 9.7	25.64	4.59-142.86	***0.001***
BD-IPMN vs. group B	18.4 vs. 9.7	1.31	0.35–4.95	0.98
M-IPMN vs. group B	28 vs. 9.7	1.59	0.32–7.94	0.94
MD-IPMN vs. BD-IPMN	81.2 vs. 18.4	19.6	4.57–83.33	***0.0004***
MD-IPMN vs. M-IPMN	81.2 vs. 28	16.3	3.02–88.08	***0.006***
BD-IPMN vs. M-IPMN	18.4 vs. 28	0.82	0.25–2.7	0.99

## Discussion

Our results clearly show a strong association between IPMN and CBD dilatation compared to the other pancreatic cyst types. After classifying IPMN into the three different subtypes, only MD-IPMN remained with statistically significant correlation with CBD dilatation with OR of 40 and 95% CI 7.9–200, while no correlation was seen with BD-IPMN and M-IPMN. Importantly, after adjusting for age and cholecystectomy that may by their selflead to CBD dilatation, only MD-IPMN remained significantly correlated with CBD dilatation. It is not uncommon for radiologists or other practitioners dealing with bile ducts to encounter incidental CBD dilatation. In the absence of a relevant clinical scenario where dilatation is anticipated, this may pose a challenge which usually leads to a further investigation to elucidate the cause behind this dilatation. The underlying mechanism of CBD dilatation with IPMN has not extensively examined, and it was beyond the scope of our study that was mainly primarily observational. However, proposed hypothesis beyond this association was addressed by a previous study reporting that the high viscosity of the pancreatic fluid and a change of its dynamics constitutes some kind of resistance to the bile duct flow leading to this dilatation [13]. Further studies are warranted to address the underlying mechanism beyond this association.

Strauss A et al. have found that the most important implication of finding dilated CBD in IPMN patients is that it represents the best independent predictor of malignancy in these patients with a PPV of 96.4%. The addition of CBD dilatation to the international guidelines improved malignancy detection significantly by over 60% without impairing specificity [13]. This finding needs thorough investigation as it may probably identify patients at high risk for malignant transformation. The other side of the same coin is that the differential diagnosis of incidental finding of dilated CBD, should include coexistent IPMN and should urge us to proceed with additional investigation by magnetic resonance imaging and/or EUS to uncover this potentially premalignant disease. Interestingly, there was no association with M-IPMN, although that M-IPMN include in it MD-IPMN component. Unfortunately, we could not explain this observation, as no data before has reported this association. However, this could be attributed to the higher main pancreatic duct diameter, as it was 5.5 ± 1.9 in the MD-IPMN vs. 4.8 ± 1.5 in the M-IPMN. On the other hand, given the small sample size in the M-IPMN, it might be that the association of CBD dilatation with M-IPMN has been overlooked. Thus, further larger cohort studies are warranted to confirm our findings.

The limitations of our study are the retrospective study design, and that it was conducted in a single center by one experienced endosonographist who described only a sonographic finding of dilated CBD without reporting the relation of this finding to malignancy prediction and that we had no bile fluid analysis, neither biopsy of the bile ducts was taken, thus leaving the mechanism behind this dilatation unknown.

In conclusion: We found a possible association between CBD dilatation and MD-IPMN. Further prospective multicenter study with a large cohort of patients is needed to confirm our findings, and robust experimental framework studies are needed to explore the underlying mechanism beyond this association.

## Data Availability

Data can be made available from the Corresponding Author on reasonable request.

## Abbreviations

CBD: common bile duct
IPMN: Intraductal Papillary Mucinous Neoplasms of the pancreas
MD-IPMN: main duct IPMN
BD-IPMN: branch duct IPMN
M-IPMN: mixed type IPMN
EUS: endoscopic ultrasound
CEA: Carcinoembryonic antigen
MCN: Mucinous cystic neoplasm
SCA: Serous cyst adenoma
PC: Pseudocyst
ASGE: American society of gastrointestinal endoscopy
OR: odds ratio
